# A Paleolithic bird figurine from the Lingjing site, Henan, China

**DOI:** 10.1371/journal.pone.0233370

**Published:** 2020-06-10

**Authors:** Zhanyang Li, Luc Doyon, Hui Fang, Ronan Ledevin, Alain Queffelec, Emeline Raguin, Francesco d’Errico

**Affiliations:** 1 Institute of Cultural Heritage, Shandong University, Qingdao, Shandong Province, P.R. of China; 2 Centre National de la Recherche Scientifique CNRS UMR5199 PACEA, Université de Bordeaux, Pessac, Nouvelle-Aquitaine, France; 3 Department of Structural Biology, Weizmann Institute of Science, Rehovot, Israel; 4 SFF Centre for Early Sapiens Behaviour (SapienCE), University of Bergen, Bergen, Norway; Institucio Catalana de Recerca i Estudis Avancats, SPAIN

## Abstract

The recent identification of cave paintings dated to 42–40 ka BP in Borneo and Sulawesi highlights the antiquity of painted representations in this region. However, no instances of three-dimensional portable art, well attested in Europe since at least 40 ka BP, were documented thus far in East Asia prior to the Neolithic. Here, we report the discovery of an exceptionally well-preserved miniature carving of a standing bird from the site of Lingjing, Henan, China. Microscopic and microtomographic analyses of the figurine and the study of bone fragments from the same context reveal the object was made of bone blackened by heating and carefully carved with four techniques that left diagnostic traces on the entire surface of the object. Critical analysis of the site’s research history and stratigraphy, the cultural remains associated with the figurine and those recovered from the other archeological layers, as well as twenty-eight radiometric ages obtained on associated archeological items, including one provided by a bone fragment worked with the same technique recorded on the object, suggest a Late Paleolithic origin for the carving, with a probable age estimated to 13,500 years old. The carving, which predates previously known comparable instances from this region by 8,500 years, demonstrates that three-dimensional avian representations were part of East Asian Late Pleistocene cultural repertoires and identifies technological and stylistic peculiarities distinguishing this newly discovered art tradition from previous and contemporary examples found in Western Europe and Siberia.

## Introduction

Our knowledge about the origins of symbolically mediated behaviors has substantially increased over the last twenty years. The idea of a symbolic explosion occurring in Europe 40,000 years ago [1–3], associated with the arrival of anatomically modern human populations in the region, has given way to a complex gradualist scenario [4–7]. Multiple evidence now demonstrates that behaviors generally associated with symbolic thought, such as producing abstract drawings and engravings, using pigments, wearing personal ornaments and performing complex mortuary practices, are three to ten times older than what was acknowledged two decades ago. It is also becoming clear that these practices emerged gradually among both African Middle Stone Age populations and the so-called archaic populations living in Europe and Asia [8–16]. Figurative representations were considered until recently the only symbolic manifestation for which Europe could claim precedence. This view was challenged in the last few years by the dating of 42,000-year-old calcite deposits covering animal, human, and hand stencil depictions at sites from Southeast Asia [17–19]. Furthermore, the modern human authorship of all Paleolithic paintings has likewise been questioned with the dating of calcite deposits covering hand stencils and painted signs from three Iberian caves, suggesting that these representations were made some 63,000 years ago, a time at which only Neanderthal populations were living in Europe [20]. Although repeatedly disputed [21–24], these dates were produced with the same methods applied to establish the age of the earliest Southeast Asian cave paintings [25] and were obtained following strict protocols, which apparently duly considered possible sources of error [26–28]. The carving of small figurines is, for the time being, the only artistic practice that may have originated in Europe and that could represent an innovation created by anatomically modern populations colonizing this region. The earliest known carvings consist of animal and human figurines sculpted in mammoth ivory, and were found at sites from the Swabian Jura, Germany, in layers containing Early Aurignacian artifacts and dated to *circa* 40–38 ka [29,30]. For vast regions of the world, however, it remains unclear when the production of three-dimensional representations became a part of the cultural repertoire of prehistoric groups, and whether this happened independently or through diffusion from a point of origin.

Here we report the discovery of a diminutive carving, depicting a standing bird, found at the Paleolithic site of Lingjing, Henan, China. Careful consideration of the site’s research history and stratigraphy, of the cultural remains it yielded in association with the figurine and from other archeological layers, as well as of the numerous radiometric ages obtained from the associated archeological items argues in favor of a Late Paleolithic origin for the carving, with a probable age of 13,500 years. The exceptional state of preservation of the figurine, unmatched by comparable Paleolithic carvings, and the application of state-of-the-art methodology to its study allowed us to reconstruct in detail the technology and evaluate the skills that led to its manufacture. Our results highlight peculiar features that make this carving the first known instance of an original artistic tradition, i.e., a body of technical, thematic, and stylistic traits applied to the production of symbolic artifacts shared by a society and transmitted to new generations of artists [31].

### Archeological context and dating

**Fig 1:**
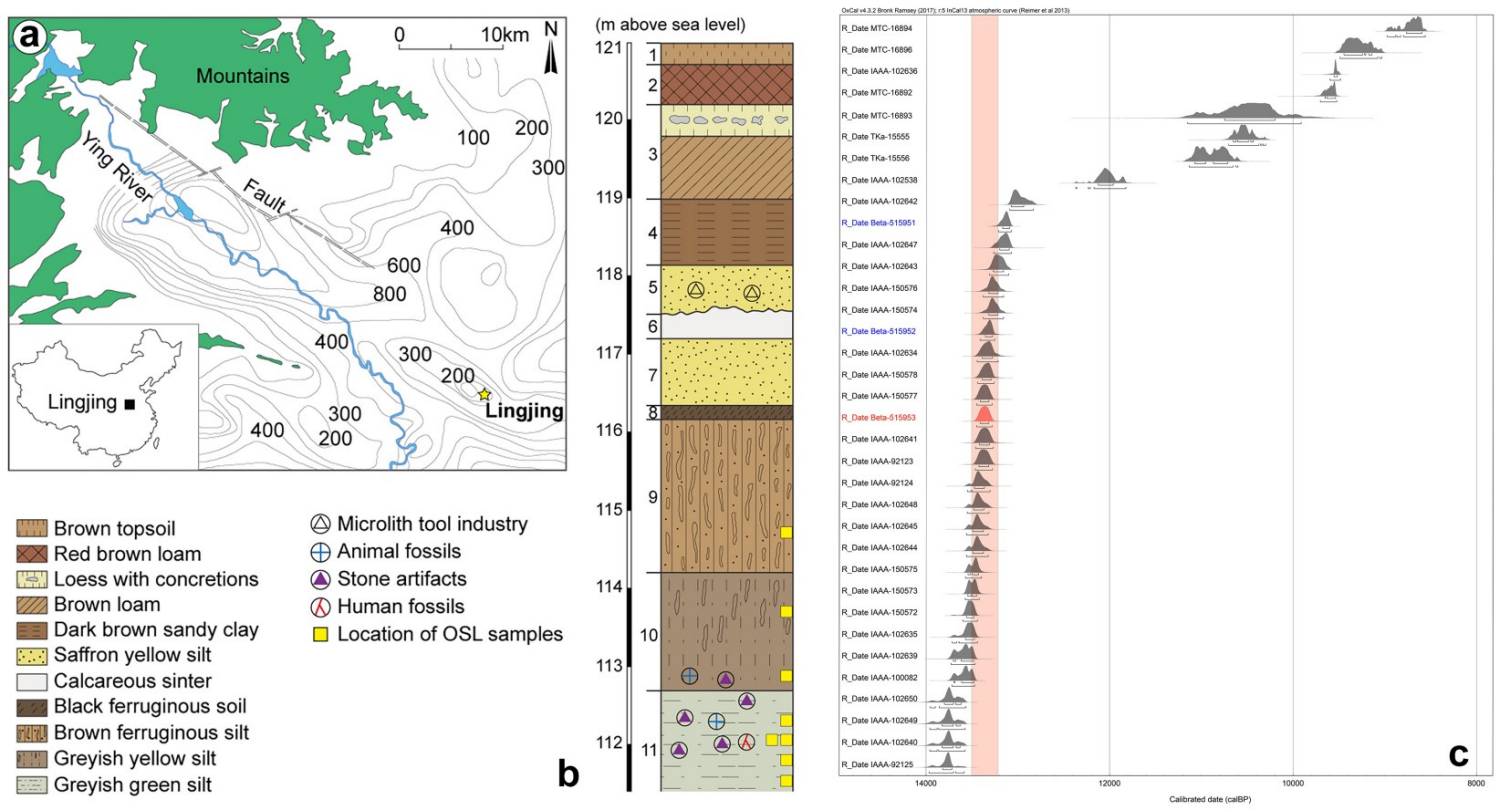
Localization and stratigraphy of the Lingjing site and ^14^C dating for the bird carving archeological context. (A) Location of Lingjing (Henan, China). (B) Stratigraphy of the sedimentary sequence with indication of main findings. (C) Calibrated ^14^C ages obtained on burnt bone, charcoal, and charred residues present on pottery sherds from the context in which the carving was recovered (see Table 1 for details). The red bar identifies the age of a burnt bone sample bearing traces of modification similar to those recorded on the carving.

Lingjing is an open-air site located in Henan, 120 km south of the Yellow River (Fig 1a). This water-lain deposit was discovered in 1965 [32,33] and excavated yearly from 2005 to 2018 under the direction of one of us (LZ). The excavation, extending over a surface of 551 m^2^, identified eleven layers, numbered from 1 at the top to 11 at the bottom, within a 9 m deep sedimentary sequence (Fig 1b). Seven layers contain archeological remains. The uppermost layers 1–4 are Holocene in age; they were identified over the entire excavated surface but were associated with archeological material only along the northern edge of the site, i.e., >50 meters north of the area where layer 5 was identified in the stratigraphy. Layers 1–4 yielded a few dozen isolated, fine pottery sherds that could not be refitted with one another. Their outer surface bears decors that allow their cultural attribution to periods spanning from the Yangshao Neolithic (~6.5–5 ka BP) to the Shang-Zhou Bronze Age (~4–2.5 ka BP). Neither stone tools nor faunal remains were recovered from these layers. Layer 5 and sediments originating from it (see below) included artifacts reflecting an occupation spanning from the LGM to the Younger Dryas. Layers 6 to 9 are sterile. Layers 10 and 11, attributed to the early Late Pleistocene, were dated by OSL between 99 ka and 118 ka [34]. Both layers yielded lithic artifacts and faunal remains [35]. Two incomplete human skulls were also found in layer 11. They bear a mosaic of morphological features interpreted as indicating both regional continuity and interregional population dynamics [33,36]. Analysis of faunal remains from layer 11 identified the earliest known evidence for pressure flaking [37], the first bone retouchers from East Asia [38], and the use of metapodials as organic soft hammer for marrow extraction [39]. Two weathered bone fragments bearing parallel engraved lines and traces of ocher were also found in this layer [15].

**Fig 2:**
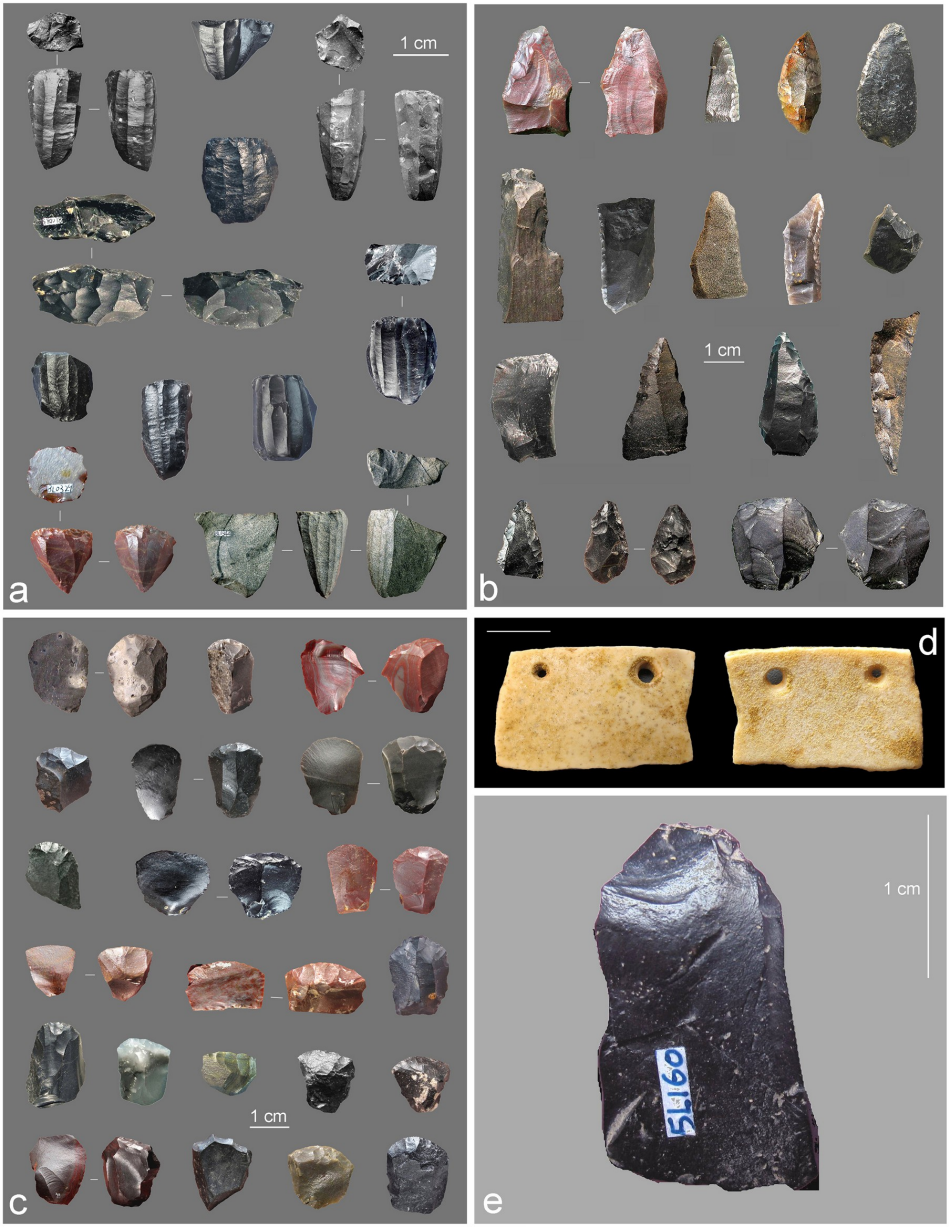
Archeological remains associated with the bird carving. (A) Selection of microblade cores. (B) Retouched tools including scrapers, burins and points. (C) End-scrapers. (D) Ostrich egg shell pendant. (E) High quality black chert flake from layer 5. Scales = 1 cm.

**Fig 3:**
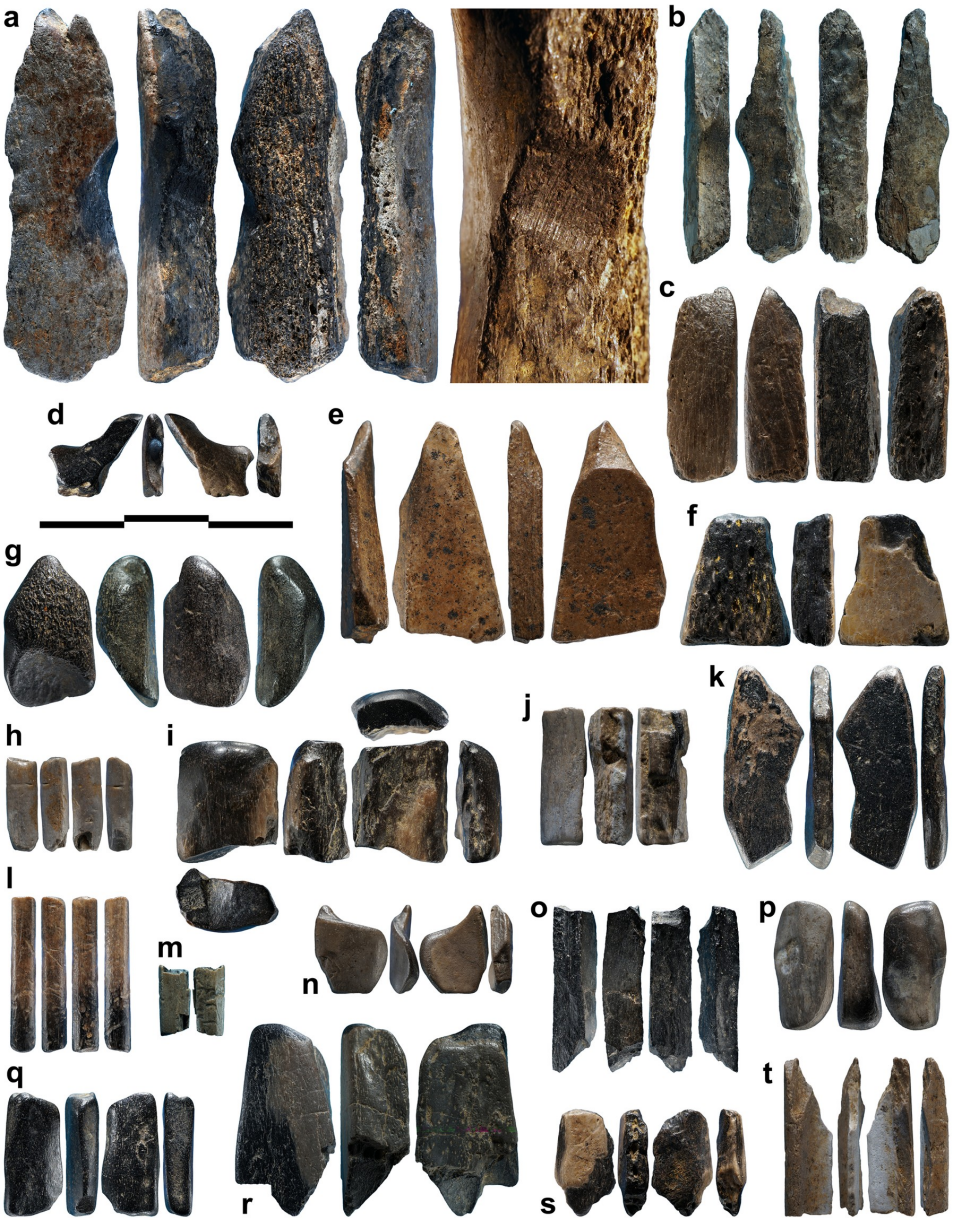
Burnt bone fragments associated with the bird carving. (A) Fragment dated by ^14^C bearing traces of gouging (close up at the right) comparable to those recorded on the carving. (B-T) Other fragments, some of which bearing traces of modification (see Table 2 for details). Scale = 1 cm.

When one of us (LZ) excavated the site in 2005, he found that most of layer 5 had been removed by well diggers in 1958 and that only a small portion of this layer was still present in the stratigraphic profile near the southern limit of trench T1. The layers overlaying the remnants of layer 5 were completely sterile in this area. The excavation of the intact portion of layer 5 yielded a small amount of quartz tools, high quality black chert microliths, and pottery sherds. During the 2008 and 2013 excavation campaigns, the spoil heap left by the well diggers in 1958 was identified less than 10 m away from the water cistern built over the spring opening, which is located near the southern edge of the excavated area. Water sieving of these sediments produced a rich microcore and microblade industry made of high-quality black chert, a raw material only found in layer 5, few pottery sherds, burnt and unburnt faunal remains, charcoal, ostrich egg shell fragments, a perforated ostrich egg shell pendant (Figs 2 and 3), and the bird figurine described in the present study. The pottery sherds found in the intact remnants of layer 5 and in the spoil heap differ greatly from those recovered from Holocene contexts i.e., layers 1–4. They are thick, crude, simple in shape, with plain surfaces, very fragile, and fired at low temperatures [40,41]. The lithic assemblage from layer 5 is dominated by pyramidal type microblade cores, followed by boat-shaped and wedge-shaped cores [41–43]. Retouched tools include in decreasing order short end-scrapers, scrapers, and burins. Analysis of this assemblage identified consistencies in raw material use (Fig 2e), technology and typology supporting the syndepositional nature of the assemblage and striking similarities with Late Glacial industries from Northern China [40–43]. Aside from the few ostrich eggshell fragments, the faunal assemblage found in association with the figurine contains 215 remains consisting of 80 unidentifiable, blackened bone fragments, a quarter of which was radiocarbon dated (see below), as well as 135 fragmentary equids and bovids molars.

Burnt bones, charcoals and charred residues from the pottery sherds recovered in the spoil heap in which the bird carving was found were radiocarbon dated (Fig 1c, Table 1) at three dating laboratories: the Institute of Accelerator Analysis Ltd., Kawasaki City, Kanagawa, Japan (IAAA), the University of Tokyo Radiocarbon Laboratory (TKa), and the Beijing University Radiocarbon Laboratory (MTC), China [40–43]. The thirty-six available ages, three of which obtained in the framework of this study (see below), were calibrated with the OxCal 4.3.2 online software [44] using the IntCal13 atmospheric calibration curve [45]. With the exception of a single old age obtained from a charcoal sample (IAAA-100080: 28,690±120 ^14^C years), not included in Fig 1c, two distinct sets of ages are identified by the dated material. The first set, which includes all ages obtained on burnt bone and charcoal, ranges between ~13.8 and ~13.0 ka cal BP. Considering the age of Northern Chinese sites with comparable microblade assemblages, this set covers the time span for the occupation of the site by Late Glacial hunter-gatherers bearing microlithic technologies. The ages of the second set, all obtained from charred residues present on pottery sherds, range from ~12.1 to ~8.6 ka cal BP. This last set probably reflects two successive occupations by ceramics users, centered around 10.3 ka cal BP and 9.2 ka cal BP [40].

**Table 1:** Calibrated ^14^C ages obtained on burnt bone and charcoal from the sediment in which the bird carving was found.

Lab ID	Material	14C age	Error	Calibrated date (1σ)			Calibrated date (2σ)			References
				From	To	Probability	From	To	Probability	
Beta-515951	BB	11290	30	13165	13090	68.2%	13211	13070	95.4%	This study
Beta-515952	BB	11470	30	13353	13275	68.2%	13412	13251	95.4%	This study
Beta-515953*	BB	11520	40	13410	13315	68.2%	13448	13279	95.4%	This study
IAAA-92123	BB	11530	50	13424	13317	68.2%	13463	13276	95.4%	40–43
IAAA-92124	BB	11590	50	13473	13364	68.2%	13500	13300	90.7%	40–43
							13548	13510	4.7%	40–43
IAAA-92125	BB	11940	40	13820	13719	68.2%	13967	13701	87.9%	40–43
							13678	13585	7.5%	40–43
IAAA-100080	CH	28690	120	33050	32593	68.2%	33310	32313	95.4%	40–43
IAAA-100082	BB	11760	40	13611	13480	64.7%	13719	13470	95.4%	40–43
				13696	13685	3.5%				40–43
IAAA-102634	BB	11480	50	13390	13276	68.2%	13443	13214	95.4%	40–43
IAAA-102635	BB	11720	50	13571	13472	68.2%	13641	13440	89.9%	40–43
							13715	13664	5.5%	40–43
IAAA-102636	CM	8570	40	9553	9517	68.2%	9600	9483	95.4%	40–43
IAAA-102538	CH	10270	40	12123	11959	68.2%	12169	11821	93.8%	40–43
							12232	12211	1.1%	40–43
							12367	12357	0.5%	40–43
IAAA-102639	CH	11760	50	13618	13481	59.6%	13726	13466	95.4%	40–43
				13703	13678	8.6%				40–43
IAAA-102640	CH	11930	50	13825	13705	59.1%	13864	13574	86.8%	40–43
				13672	13629	9.1%	13958	13879	8.6%	40–43
IAAA-102641	BB	11520	50	13416	13310	68.2%	13460	13270	95.4%	40–43
IAAA-102642	CH	11120	50	13071	12935	68.2%	13089	12830	95.4%	40–43
IAAA-102643	BB	11370	50	13268	13155	68.2%	13307	13099	95.4%	40–43
IAAA-102644	CH	11610	50	13490	13374	68.2%	13559	13322	95.4%	40–43
IAAA-102645	BB	11610	50	13490	13374	68.2%	13559	13322	95.4%	40–43
IAAA-102647	CH	11300	50	13198	13094	68.2%	13263	13070	95.4%	40–43
IAAA-102648	CH	11600	50	13480	13370	68.2%	13550	13314	95.4%	40–43
IAAA-102649	BB	11930	50	13825	13705	59.1%	13864	13574	86.8%	40–43
				13672	13629	9.1%	13958	13879	8.6%	40–43
IAAA-102650	CH	11920	50	13800	13702	51.8%	13856	13566	90.7%	40–43
				13678	13617	16.4%	13957	13898	4.7%	
IAAA-150572	BB	11710	40	13558	13479	68.2%	13599	13441	95.4%	40
IAAA-150573	BB	11660	40	13498	13449	39.1%	13574	13421	95.4%	40
				13545	13507	29.1%				
IAAA-150574	BB	11430	40	13316	13216	68.2%	13377	13155	95.4%	40
IAAA-150575	BB	11639	40	13495	13427	53.3%	13570	13395	95.4%	40
				13543	13515	14.9%				
IAAA-150576	BB	11430	40	13316	13216	68.2%	13377	13155	95.4%	40
IAAA-150577	BB	11520	40	13410	13315	68.2%	13448	13280	95.4%	40
IAAA-150578	BB	11490	40	13386	13291	68.2%	13437	13259	95.4%	40
TKa-15555	CM	9330	60	10603	10486	50.1%	10703	10372	92.4%	40
				10653	10621	9.6%	10326	10299	2.2%	
				10461	10433	8.4%	10354	10341	0.8%	
TKa-15556	CM	9530	70	10869	10711	37.5%	11133	10652	93.8%	40
				11071	10950	30.7%	10623	10601	1.6%	
MTC-16892	CM	8630	50	9630	9536	66.5%	9703	9523	95.4%	40
				9652	9549	1.7%				
MTC-16893	CM	9250	210	10746	10196	68.2%	11148	9911	95.4%	40
MTC-16894	CM	7880	50	8764	8595	68.2%	8797	8559	78.1%	40
							8976	8881	11.0%	
							8869	8827	6.3%	
MTC-16896	CM	8310	90	9445	9240	57.7%	9489	9077	93.5%	40
				9175	9140	7.7%	9055	9033	1.9%	
				9220	9206	2.8%				

BB: Burnt bone; CH: Charcoal; CM: Carbonized material from pottery sherds

* Object bearing traces of manufacture similar to those on the bird carving

In order to estimate the age of the bird figurine, three new burnt bone samples, one of which bearing evidence of gouging, an anthropogenic modification observed on the bird carving (see below), were sent to the Beta Analytic Testing Laboratory, Miami (FL, USA). All of them were analyzed following the ISO/IEC 17025:2005 Testing Accreditation PJLA #59423 protocols. Charred bone pretreatment with alkali allowed for the extraction of collagen. All work was done at Beta facilities in four in-house NEC accelerator mass spectrometers and four Termo IRMSs. The “Conventional Radiocarbon Age”, calculated using the Libby half-life i.e., 5,568 years, was corrected for total isotopic fraction, and was used for calendar calibration. Conventional Radiocarbon Ages and sigma were rounded to the nearest 10 years and are reported as radiocarbon years before present, i.e., before 1950. When counting statistics produced sigma lower than 30 years, a conservative ± 30 BP was cited for the results. δ^13^C values were obtained on the material itself, not on the AMS δ^13^C.

The three burnt bone samples selected for radiocarbon dating yielded sufficient amount of collagen within the expected value required for passing the quality assurance tests. All three ages fall within the first cluster previously identified and range between 13,4 and 13,1 ka cal BP (Fig 1c, Table 1). The age of the bone bearing a deep notch produced by gouging is 11,520 ± 40 (Beta-515953), which corresponds to an age of 13,448–13,279 cal BP (95.4%). It is noteworthy that twenty-one of the twenty-eight (75%) ^14^C ages from the first set statistically overlap (95%) the age obtained on the modified bone (Fig 1c). The coherent results obtained for the ages of bone and charcoal samples indicate that both the faunal remains and the fire fuel, i.e., the charcoals, are of the same age, which rules out the possibility that the craftsman would have selected a sub-fossil fragment to burn and to carve the figurine [46].

Although the bird figurine was found in a spoil heap, a number of contextual observations argue in favor of its Paleolithic origin. First, the spoil heap did not contain Neolithic or Bronze Age cultural remains. Likewise, no archeological remains attributed to Paleolithic ages were found in layers 1–4; these latter layers were archeologically sterile in the area where remnants of layer 5 and the associated spoil heap were identified. The distance separating the areas containing Neolithic/Bronze Age and LGM/Younger Dryas archeological material is too important, i.e., >50 m, to suggest the carving percolated into layer 5 from overlying more recent layers. The absence of evidence suggesting the mixing of both assemblages, i.e., from layer 5, on the one hand, and from layers 1–4 on the other, is further supported by the differences in the manufacture, decoration, and ages of the ceramic remains. While the sherds from layer 5 are crude, without decoration, and fired at low temperature, those from layers 1–4 are fine and bear stylistic features that allow their cultural attribution to the Yangshao Neolithic and Shang-Zhou Bonze Age. It is well-known that crude pottery appears in the Chinese archeological record at the end of the Upper Paleolithic and in Epipaleolithic contexts. The oldest known occurrences appear *circa* 20 ka BP in South China [47–49] and *circa* 12 ka BP in North China [40,50]. Should the pottery sherds from layer 5 have had a Neolithic/Bronze Age origin, their expected ages would have ranged between 7 and 5 ka BP, not 12 to 9 ka BP. Finally, although the figurine could date to any of the three episodes of the human occupation identified by the ^14^C ages, none of the numerous dated faunal remains falls into the timeframe of the two most recent proposed human occupations, i.e., between 11 and 10 ka BP or between 9.6 and 8.7 ka BP; their radiocarbon ages are exclusively comprised between 13.8 and 13 ka BP regardless of the testing laboratory to which they were submitted for dating. Moreover, the figurine and associated faunal remains feature a similar color range and patina, which argues in favor of their syndeposition. These results and contextual information indicate that the most probable age of the figurine corresponds to that of the directly dated faunal remain bearing evidence of gouging, i.e., within the ~13.4–13.2 ka cal BP time interval.

## Materials and methods

The Lingjing bird carving and the associated archeological material analyzed in this study are curated at the Henan Provincial Institute of Cultural Relics and Archaeology, Zhengzhou, Henan Province, China (Repository ID: 09L5鸟-01). No permits were required for the described research, which complied with all relevant regulations. The Lingjing bird carving was 3D scanned using a General Electrics (GE) Vtome x|s microtomography housed at the PLACAMAT facilities, Bordeaux University. The figurine was scanned with the beak facing downward to minimize artifacts produced when X-rays are tangential to the surface. The acquisition was done with a 180 kV X-ray nanofocus tube and the following beam parameters: 100 kV and 200 μA. The scan was performed at a cubic voxel size of 11.5 μm. A copper filter of 0.1 mm was used and 1875 projections were taken at 360°. The reconstruction of the volume was performed with the Datos | Rec software of GE. 3D visualizations were then performed within the Avizo 9.1 (FEI) workspace. A simplified 3D surface was also generated (S1 Data) with the MiKTeX Console 2.9.7076.

The object was photographed with a Sony E 30 mm with a macro-lens, and examined and photographed with a motorized Leica Z6 APOA equipped with a DFC420 digital camera linked to LAS Montage and Leica Map DCM 3D computer software. Images showing various aspects of the figurine were imported into Adobe Illustrator and used to make a tracing of the areas bearing traces of manufacture identified under the microscope. This tracing was compared to the original specimen under a microscope and corrected as required. High-resolution surface topography of selected areas was obtained with a Sensofar S neox confocal microscope driven by SensoScan 6 software (Sensofar) in order to better visualize and characterize traces of manufacture and use wear. Data acquired from the various imaging methods were compared to establish the technique of manufacture used and the orientation of the motion on each identified area. Distinction between traces of manufacture and alterations resulting from use was based on diagnostic features identified on ethnographic, experimental and archeological bone items [51–62]. When two or more techniques were identified on the same area, partial obliteration of the original anthropogenic modifications by traces generated through the subsequent application of another technique clarified their chronological ordering.

## Results

The figurine depicts a small standing bird (length 19.2 mm, width 5.1 mm, height 12.5 mm). The subject’s morphology and proportions, i.e., short head and neck, robust, rounded bill and long tail, are reminiscent of Passeriformes. Passeriformes is an order that encompasses more than half of all known extant bird species. Unfortunately, the lack of minute details on the figurine prevents a more precise identification. Aside from the ostrich eggshell fragments found in the spoil heap, no avian remains were identified in the faunal assemblages of any archeological layers at Lingjing, i.e., layers 1–4, 5, 10, and 11. Nonetheless, our identification of the carving as representing a passerine is fully compatible with the paleoecology of the species comprised in this order as they were, and are, found on virtually all continents and climatic settings, including present-day and Late Pleistocene China [63,64]. In lieu of the passerine short legs, a large, rectangular pedestal allows the figurine to stand in the upright position (Fig 3, S1 Data). The oversized tail prevents the object from tilting forward. The lateral aspects of the body are flat and the wings are not represented.

**Fig 4:**
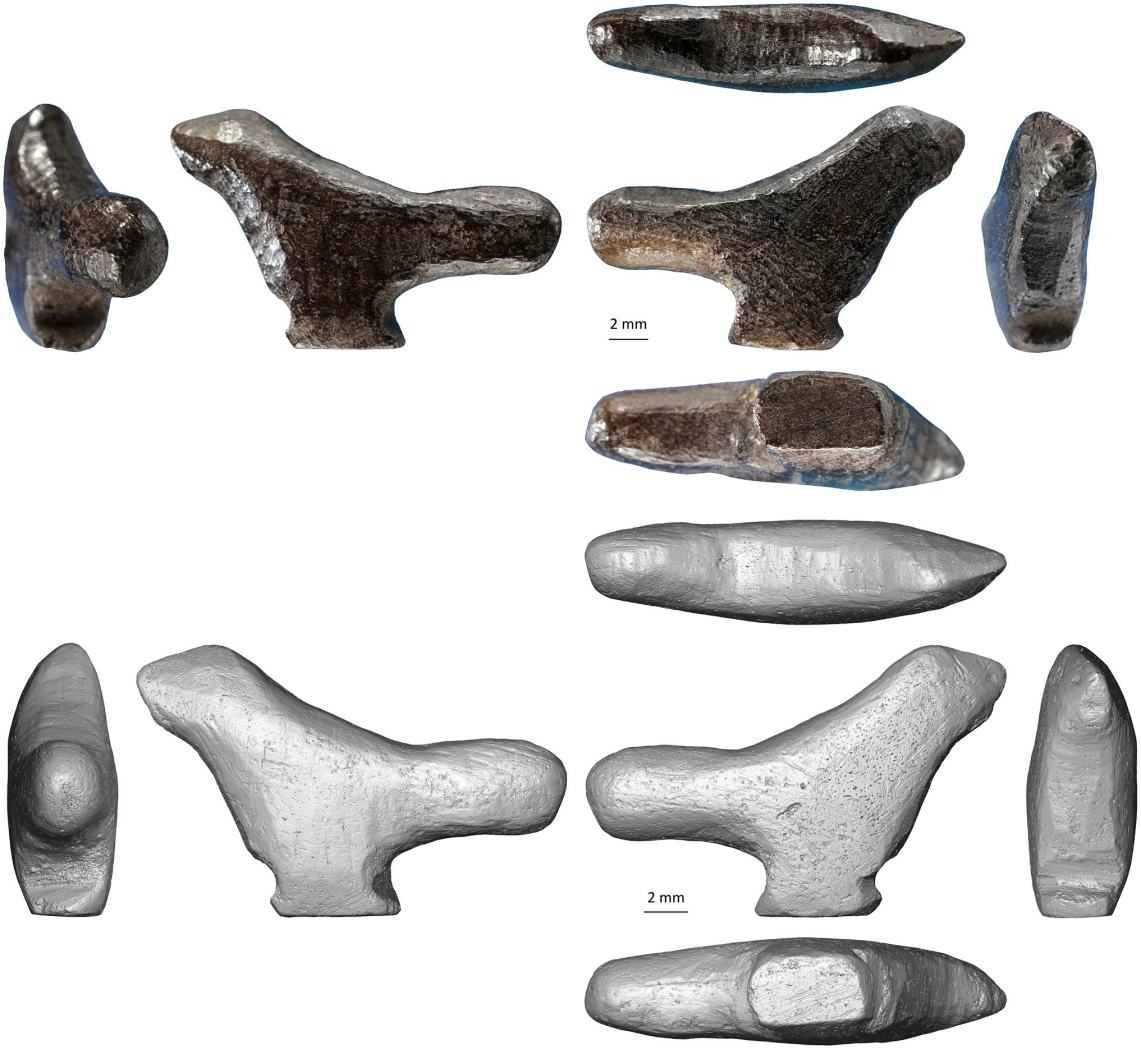
Lingjing bird carving. (A) Photographs of the six aspects of the carvings. (B) 3D renderings of the carving obtained by CTscan. Scales = 2 mm.

Openings left on the surface by capillaries and vascular canals demonstrate the use of cortical bone for the manufacture of the figurine (Fig 4a–4d, S1 Video). Microtomography reveals a densely vascularized fibrolamellar complex without evidence of remodeling. The orientation of the bone structure corresponds to the bill-tail axis of the carving (S1 Video). The lack of lamellated layers and the presence of the same pattern of vascularization throughout the figurine suggest that the bone outermost and innermost cortex were removed when carving the object. The presence of transverse layers of demineralized bone is consistent with the action of osteolytic bacteria [65–67] (Fig 4e). Increased bone density at the surface of the object indicates the infilling of mineral deposits following physicochemical and biochemical alterations [65,68,69] after the action of microorganisms. Reticular fibrolamellar bone tissue is found in birds, large dinosaurs and in long bones of fast-growing juvenile mammals such as herbivores [70–75]. Considering the orientation of the bone structure and the figurine dimensions, the bird was likely carved from a diaphyseal fragment of a medium-size mammal limb bone.

The bone figurine as well as other worked and unworked osseous fragments found in the spoil heap are completely blackened (Fig 3, Table 2). Recent and heat fractures present on some specimens illustrate the extent of the process in cross-section. The color gradient ranges from a dark brown, almost black, outer layer measuring between 0.2 mm to 0.8 mm in thickness to dark black at the center. None of the original bone coloration remains visible on the specimens. Although the staining of bones could result from a variety of processes [76], the observed color gradient and the density of the bone fragments suggest they might have been subjected to a controlled heat treatment. Exposure to open flame, and contact with calcitic ash, may cause the bone to crack, shrink and deform. However, the recognition of histological structure remains achievable when bone is burnt at less than 600°C [77–85]. Experimental data highlights that the temperature of cremation, the duration of heat exposure, and the availability of oxygen and organic compounds in the environment play key roles in modifying the aspect and structure of osseous remains [86]. Based on the color gradient visible in cross-section, and considering that microtomography reveals an intact histological structure, the faunal fragments were most likely heated from 1 to 3 hours in an anaerobic environment at a temperature ranging between 300 and 500°C. Controlled experiments are required, however, to reproduce this treatment accurately and assess its effects on easing the carving of osseous raw material.

**Table 2:** Technological and morphometric data on burnt bone fragments associated with the bird carving.

Figure	Maximum Length	Maximum Width	Maximum Thickness	Cortical Thickness	Human modification	Modification type	Burnt	Fracture Type
Fig 3a	43.39	14.65	9.67	6.04	Yes	Fl, Go, Po	y	
Fig 3b	29.01	7.82	5.28		Yes	Po, St	y	Recent
Fig 3c	21.21	7.56	6.67		No		y	
Fig 3d	13.31	7.61	3.64		Yes	Ab	y	
Fig 3e	24.53	11.98	4.04		No		y	
Fig 3f	15.3	12.61	5.52	5.17	Maybe	Fl, Go(?), St	y	
Fig 3g	17.92	10.45	6.81		No		y	
Fig 3h	10.99	3.7	3.21		Yes	Cm, No, St	y	
Fig 3i	14.43	11.61	6.64		Yes	No, St	y	
Fig 3j	15.23	6.21	4.75		No		y	
Fig 3k	24.1	9.46	3.17		Yes	Ab, Po	y	
Fig 3l	16.84	2.87	2.79		Yes	Po, Sa(?)	y	Old
Fig 3m	10.25	4.8	3.23		No		y	
Fig 3n	10.46	8.77	3.07	2.13	No		y	
Fig 3o	19.03	5.09	4.2		Yes	Pf, Re	y	
Fig 3p	14.81	7.24	4.78		No		y	
Fig 3q	14.3	6.81	3.81		No		y	
Fig 3r	21.81	11.79	6.56	6.3	Yes	Fl, Po	y	Heat
Fig 3s	12.04	6.91	3.38		Maybe	Fl	y	
Fig 3t	18.74	5.75	2.93		No		y	Recent

Ab: Abrading; Cm: Cut mark; Fl: Flaking; Go: Gouging; No: Notching; Pf: Pressure flaking; Po: Polishing;

Re: Retouching; Sa: Sawing; St: Striations

**Fig 5:**
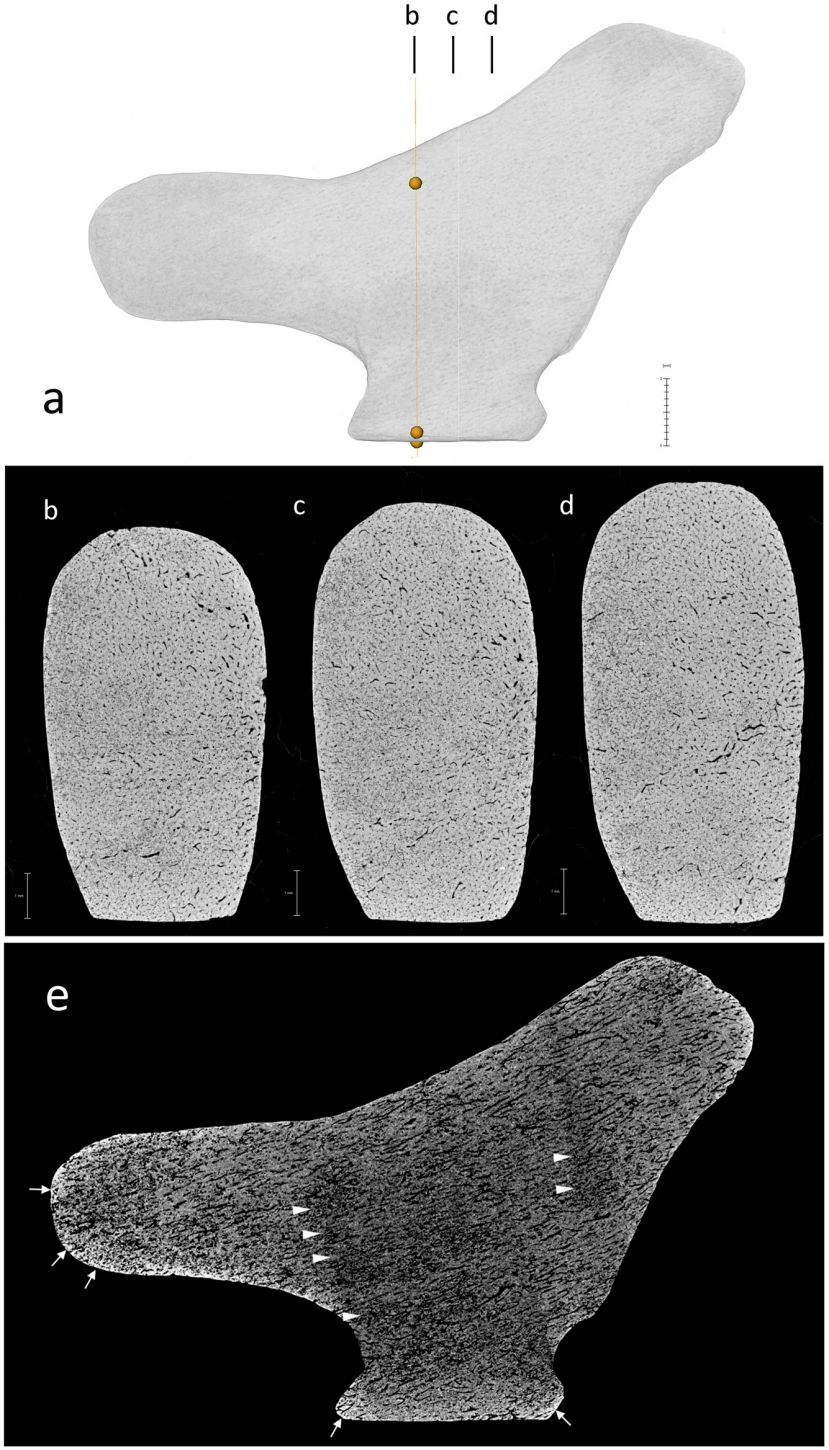
Microtomography of the Lingjing bird carving. (A) Location of the three sections. (B-D) Transverse sections showing that inner cortical bone was used to carve the object and that the bill-tail axis was oriented along the bone structure. (E) Longitudinal section showing diagenetic alterations. Increase density near the surface (arrows) indicate probable infilling of mineral deposits following physicochemical and biochemical alteration. Transversal layers of demineralized bone (arrow heads) suggesting the action of osteolytic bacteria. Scales = 2 mm.

**Fig 6:**
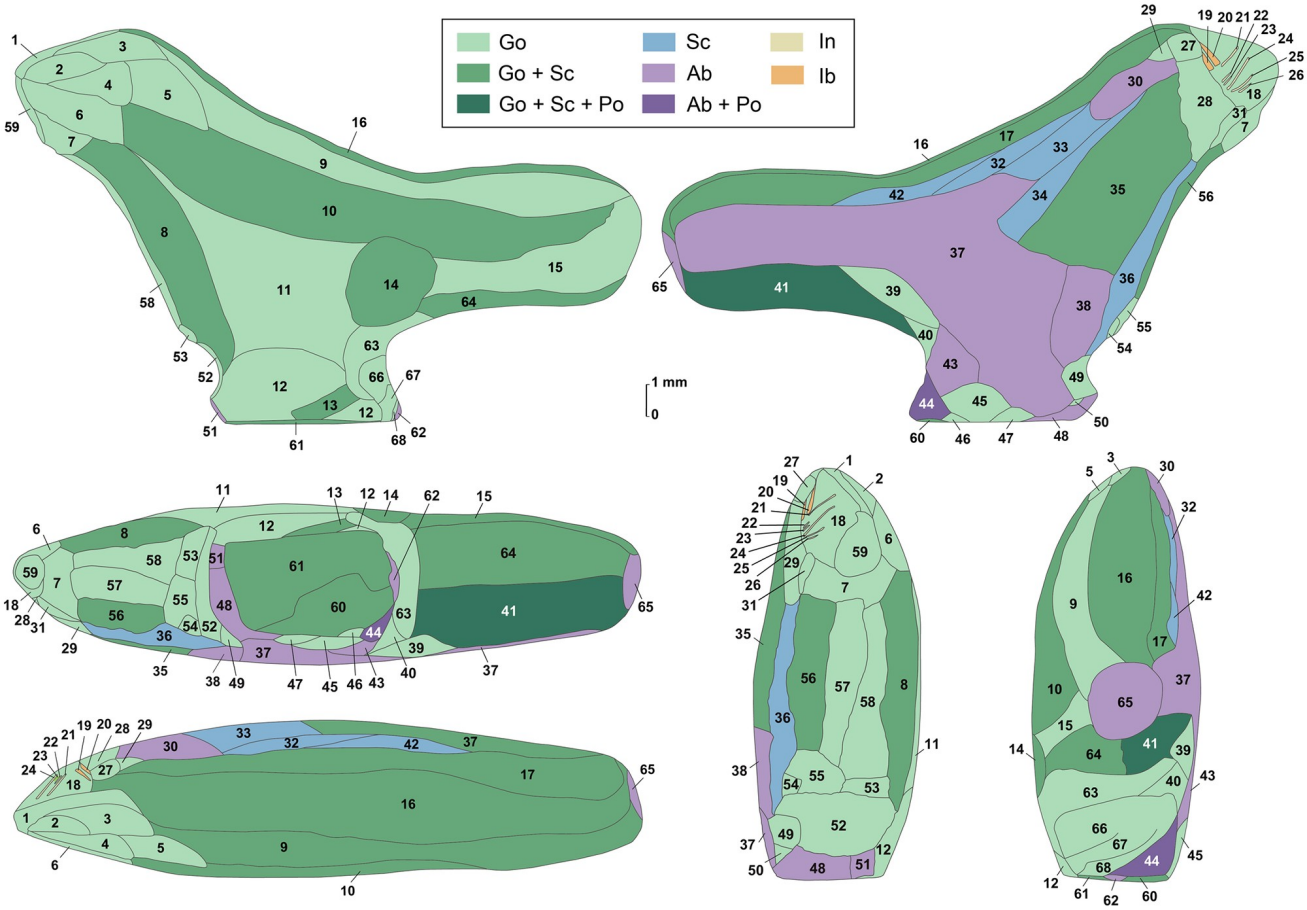
Tracings of the six aspects of the Lingjing bird carving with the technique used to manufacture each area. Ab: Abrading; Go: Gouging; Ib: Incising with a burin; In: Incising; Po: Polishing; Sc: Scraping. Numbers refer to Table 3. Scale = 1 mm.

To study the manufacturing process of this exceptional object, we applied a novel approach consisting in surveying diagnostic traces of manufacture and post-depositional modifications by multifocus and confocal microscopy, while using high-resolution microtomography to detect edges between worked areas. We recorded sixty-eight areas distributed over the entire surface of the object, each corresponding to distinct carving episodes leading to the shaping of the figurine (Fig 5, Table 3). For each area, we documented the manufacturing technique applied and, when possible, the orientation of the gesture. In thirteen cases, we were able to identify the superimposition of techniques. Gouging was applied with the robust edge of a stone tool, such as a burin spall, producing flat facets covered by chatter marks, i.e., undulations perpendicular to the direction of the tool motion generated by pressure changes (Fig 6a). Prolonged use of a burin in a gouging activity may result in the microchipping of its flat edge, which can then produce uninterrupted superficial striations running parallel to the gouging motion and keeping a constant depth on the undulations of the chatter marks. Abrading was performed by displacing the object on an abrasive grindstone which resulted in broad, spindle-like grooves of variable depths (Fig 6b). Scraping was achieved with retouched and unretouched lithic cutting edges resulting in groups of shallow grooves featuring internal parallel striations (Fig 6c). Incisions were done either by deeply engraving the surface with the dihedral-shaped tip of a stone tool, or by superficially marking the bone with a sharp point (Fig 6d).

**Table 3:** Location and manufacturing technique applied to each area of the bird figurine.

Area N°	Techniques		Visible on aspect:						Area N°	Techniques		Visible on aspect:					
	1st	2nd	Right	Left	Top	Bottom	Front	Rear		1st	2nd	Right	Left	Top	Bottom	Front	Rear
1	Go			•	•		•		35	Go	Sc	•			•	•	
2	Go			•	•		•		36	Sc		•			•	•	
3	Go			•	•			•	37	Ab		•		•	•	•	•
4	Go			•	•				38	Ab		•			•	•	
5	Go			•	•			•	39	Go		•			•		•
6	Go			•	•	•	•		40	Go		•			•		•
7	Go		•	•		•	•		41	Go	Sc, Po	•			•		•
8	Go	Sc		•		•	•		42	Sc		•		•			•
9	Go			•	•			•	43	Ab		•			•		•
10	Go	Sc		•	•			•	44	Ab	Po	•			•		•
11	Go			•		•	•		45	Go		•			•		•
12	Go			•		•	•	•	46	Go		•			•		
13	Go	Sc		•		•			47	Go		•			•		
14	Go	Sc		•		•		•	48	Ab		•			•	•	
15	Go			•		•		•	49	Go		•			•	•	
16	Go	Sc	•	•	•			•	50	Go		•				•	
17	Go	Sc	•		•			•	51	Ab			•		•	•	
18	Go		•		•	•	•		52	Go			•		•	•	
19	Ib		•		•		•		53	Go			•		•	•	
20	Ib		•		•		•		54	Go		•			•	•	
21	In		•		•		•		55	Go		•			•	•	
22	In		•		•		•		56	Go	Sc	•			•	•	
23	In		•		•		•		57	Go					•	•	
24	In		•		•		•		58	Go			•		•	•	
25	In		•				•		59	Go			•		•	•	
26	In		•				•		60	Go	Sc	•			•		•
27	Go		•		•		•		61	Go	Sc		•		•		•
28	Go		•		•	•			62	Ab			•		•		•
29	Go		•		•	•	•		63	Go			•		•		•
30	Ab		•		•			•	64	Go	Sc		•		•		•
31	Go		•			•	•		65	Ab		•		•	•		•
32	Sc		•		•			•	66	Go			•				•
33	Sc		•		•				67	Go			•				•
34	Sc		•						68	Go			•				•

Ab: Abrading; Go: Gouging; Ib: Incising with burin; In: Incising; Po: Polishing; Sc: Scraping

**Fig 7:**
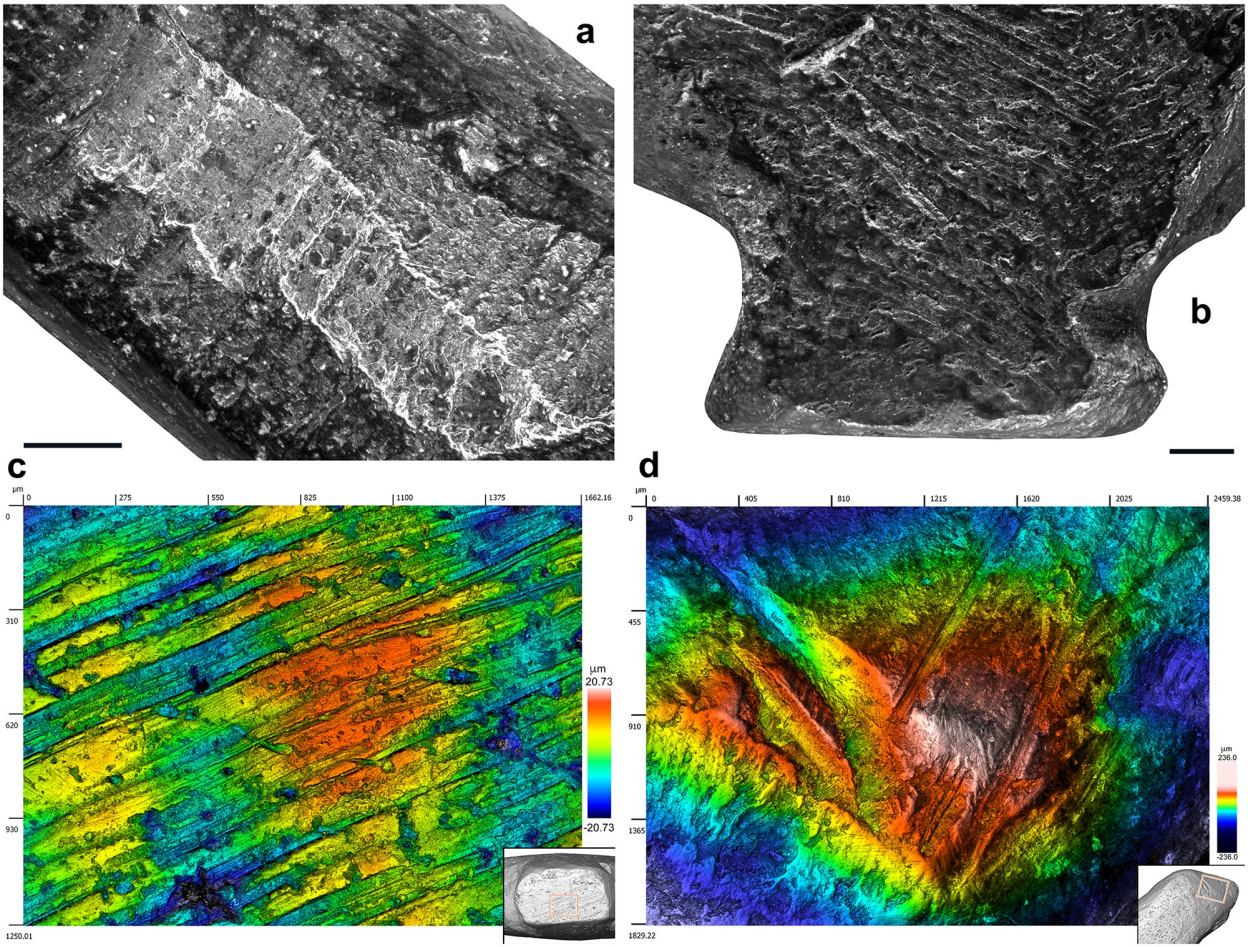
Techniques used to carve the Lingjing bird figurine. (A) Gouging used to shape the throat and breast of the bird. Scale = 1 mm. (B) Abrading applied on the right side of the figurine. Scale = 1 mm. (C) Scraping used to even out the base of the pedestal. (D) Incising on the right aspect of the head, probably to depict the eye and the bill edge, producing two converging groups of incisions, one on the left composed of two deep incisions with v-shaped sections (see Fig 6 n.19-20), the other consisting of six superficial incisions with similar internal striations demonstrating the use of the same point (see Fig 6 n. 21–26). (A-B) black and white micrographs. (C-D) 3D renderings obtained with a confocal microscope.

**Fig 8:**
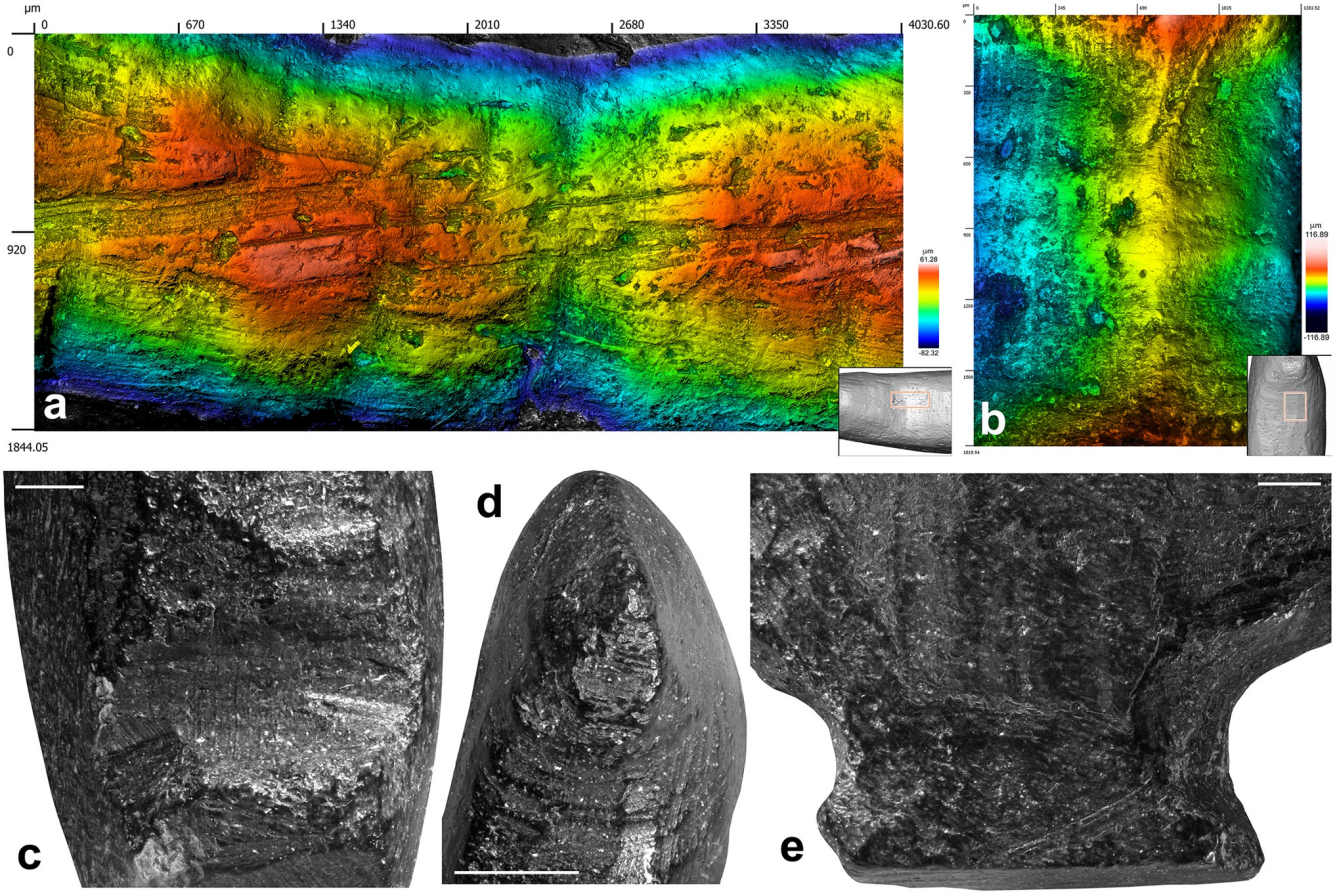
Manufacturing techniques applied to the Lingjing bird. (A) Traces of gouging followed by scraping on the bird back. (B) Traces of gouging on the bird throat leaving diagnostic chatter marks on juxtaposed facets. (C) Large notch produced by multiple vigorous gouging motions to shape the pedestal. Scale = 1 mm. (D) Gentle gouging applied to carve the head of the birds. Scale = 2 mm. (E) Superficial gouging applied to the left side of the figurine. Scale = 1 mm. (A-B) 3D renderings obtained with a confocal microscope. (C-E) Black and white micrographs.

**Fig 9:**
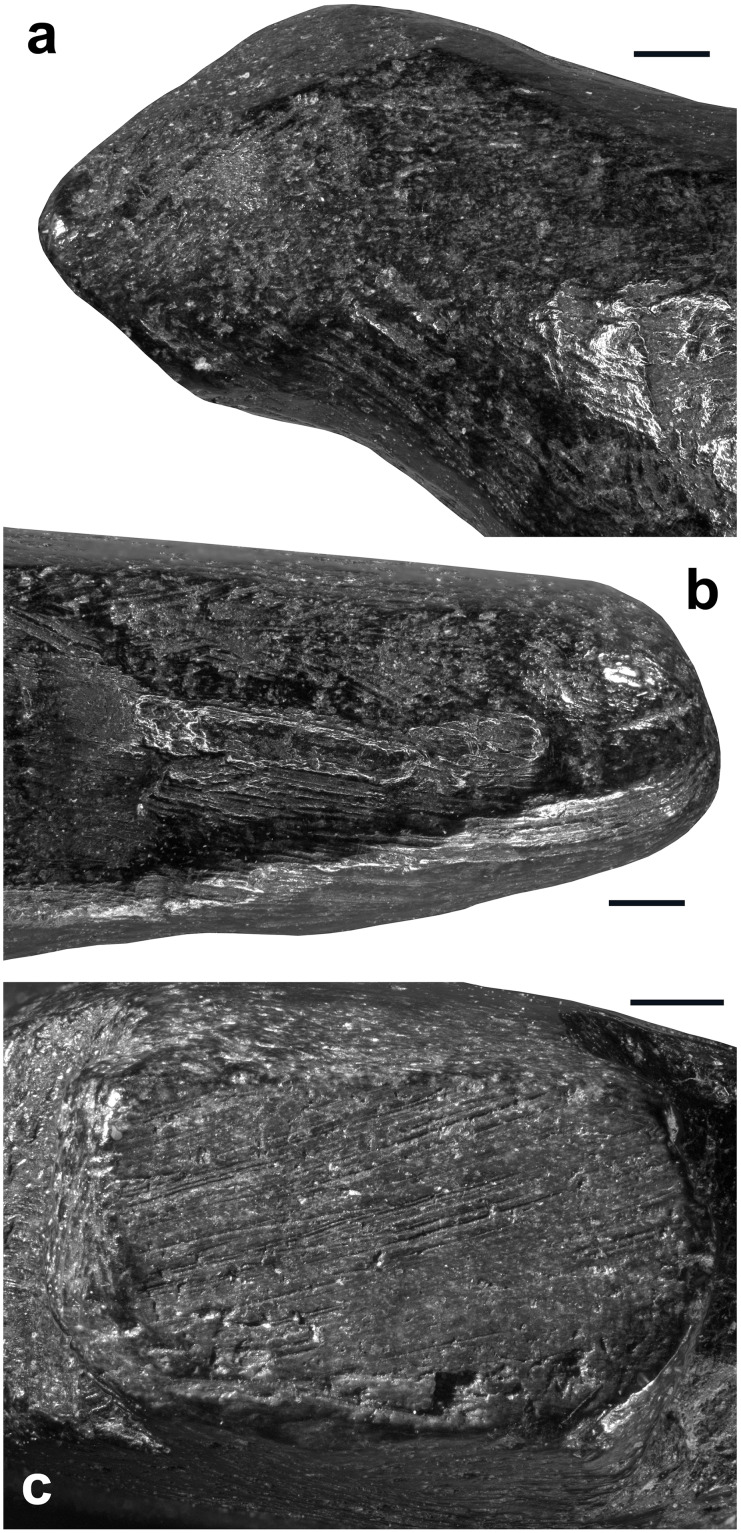
Manufacturing techniques applied to Lingjing bird. (A) Traced of scraping on the left side of the bird throat. (B) Traces of scraping on the left side of the bird tail. (C) Base of the pedestal with traces of scraping superimposed to those of gouging. (A-C) Black and white micrographs. Scales = 1 mm.

Although the initial stage of manufacture cannot be identified, it is possible that the first step entailed abrading the bone fragment. A large area on the right side of the figurine, covered by traces of abrasion on a coarse grindstone may represent the remnant of this first shaping event (Fig 6b). Gouging was used to rough out the figurine. This technique was vigorously applied to shape concave surfaces such as the throat and breast (Figs 6a and 8a, S1 Data), the back (Fig 7b, S1 Data), the undertail coverts and, in particular, the pedestal (Fig 7c, S1 Data). It was used more gently to carve the head (Fig 7d, S1 Data) and flat sides of the figurine (Fig 7e, S1 Data). Scraping was used on both sides of the throat (Fig 8a, S1 Data), the back (Figs 7b and 9a, S1 Data) and the vent (Fig 8b, S1 Data), to smooth out the chatter marks, and refine the final shape of the carving. The edges of the pedestal were carefully shaped by juxtaposing tiny facets of abrasion (S1 Data). The base of the pedestal was first carved by gouging and subsequently even out by two episodes of scraping (Figs 7c and 8c, S1 Data). The purpose of the second episode, carried out with a retouched cutting edge, may have been to slightly change the orientation of the base to ensure the bird could stand upright. On the right aspect of the head, two groups of incisions may have served to identify the bird’s eye and bill (Fig 7d). The first group is composed of two deep incisions made with the same burin. The second includes six subparallel, superficial incisions made with the same sharp point.

**Fig 10:**
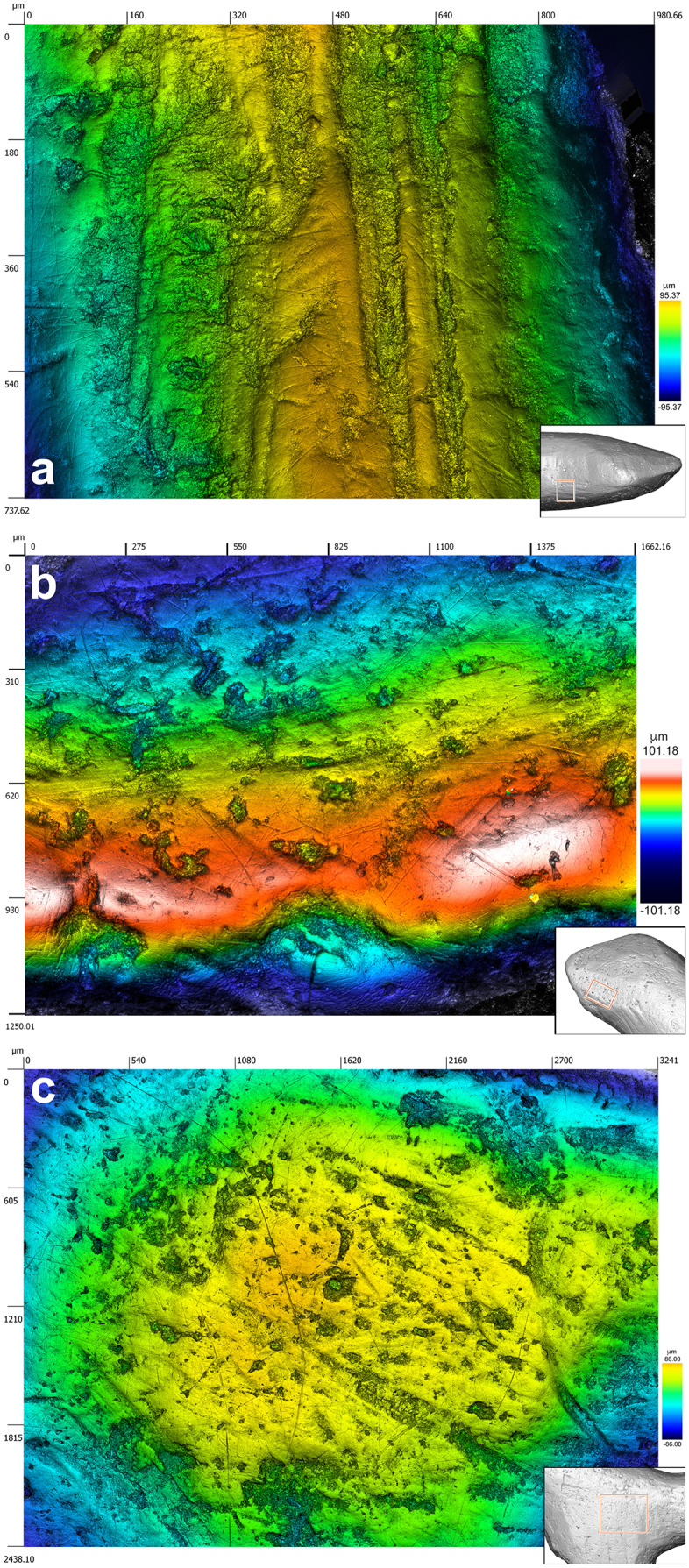
Microscopic wear. (A) Area on the back of the figurine showing traces of scraping smoothed by wear associated with randomly oriented striations. (B-C) Areas on the left side of the head (B) and the body (C) on which traces of manufacture are removed by wear associated with randomly oriented striations. (A-C) 3D renderings obtained with a confocal microscope.

At the microscopic scale, prominent features left by the manufacture are smoothed at varying degrees by an abrasion that has produced randomly oriented striations (Fig 9). The absence of such smoothing associated with randomly oriented striations of different widths on the other small blackened bone fragments from the same assemblage rules out the possibility that this wear may result from natural mechanical abrasion. The presence of sediment in some striations indicates they are ancient in origin. Based on experimental criteria, these traces differ markedly from those produced by manipulation or intentional polishing with skins or furs. They are, however, entirely consistent with use wear pattern resulting from the experimental transportation of an osseous object in a leather bag [60]. Other striations cutting into sediment deposits developed following the deposition of the object. Finally, we also recorded traces of gouging, abrading, scraping and incising on small fragments of burnt bone recovered from the same context (Fig 10, Table 2). Aside from the specimen, bearing a deep notch produced by gouging (Fig 10a, Table 2) and directly dated to 13,448–13,279 cal BP (Beta-515953: 11,520±40 BP), the assemblage also contains fragments of bone rods shaped by scraping displaying incisions perpendicular and oblique to the main axis (Fig 10, Table 2).

## Discussion

A representation is generally defined as the use of signs that stand in for, and take the place of, something else [87,88]. In the domain of artistic expressions, a representation is a type of recording in which the sensory information about a physical object, or being, is recorded in a medium. The degree to which an artistic representation resembles the object, or the being, it represents is a function of resolution. Our contention for the Lingjing figurine representing a bird is based on four lines of evidence. First, its outline, with the exception of the pedestal, almost perfectly matches that of a bird and identifies several avian anatomical features, e.g., the tail, head, bill, throat, breast, and belly. Second, the edges of the outline are modified on both aspects to enhance the anatomical features of most birds, i.e., rounded volume of the body, conical morphology of the bill, etc. Third, marks were added on the head at the location of the eye and bill. Finally, the technological analysis of the modifications present on the carving demonstrates they were deliberately produced, and the carving techniques were coherently chosen in order to highlight the anatomical features of a bird. The fact that the wings are not carved does not represent an obstacle to identifying the carving as a representation of a bird since an artistic representation is by definition an operation of subtraction, addition, and/or modification of the real world, which depends on the chosen medium, the artist’s know-how and skills, and cultural rules he/she wishes to comply to, or to transgress. Thus, the absence of wings may be explained by limitations inherent to the thickness of the bone fragment chosen to produce the carving, the diminutive size of the figurine itself, the difficulties to carve these features with the techniques and/or tools available to the artist, the stylistic canons the craftsman was conforming to, or a combination of these reasons.

Although the definition of an artistic tradition would ideally require the identification of shared technical, thematic, and stylistic traits on a number of asynchronous artistic productions, we argue that, in the case of single items, striking concomitant differences in different characterizing domains, i.e., technological, thematic, and stylistic, may reasonably be used to infer original artistic traditions. Our analysis reveals that the Lingjing artist has chosen the appropriate techniques and applied them skillfully to faithfully reproduce the distinct anatomical features of a passerine. The style of this diminutive representation is original and remarkably different from all other known Paleolithic avian figurines. Avian representations, and passerine in particular, constitute a recurring theme in Chinese Neolithic art, the oldest example being a passerine made of jade dating back to *circa* 5 ka BP [89,90]. The Lingjing bird carving predates previously known instances from this region by almost 8,500 years. The sophistication reflected by the object manufacturing process suggests this three-dimensional representation is several conceptual stages removed from the origin of a long-standing artistic tradition, extending well into the Paleolithic, that may be better characterized by future discoveries.

Even though carving and painting are generally seen as activities demonstrating the acquisition of symbolic thought, the ways in which they reify meaning in matter differ markedly. Each activity involves different spatial conceptualizations, sensorimotor experiences, analogical reasonings, and skill-learning processes [91–93]. Pigment preparation and application obviously play a key role in painting. At times, particular morphological features of the canvas, e.g., natural protuberances or concavities of a cave wall, might have been exploited to enhance the perspective of a representation. Carving a figurine, on the other hand, requires the combination of different techniques, e.g., scraping, grinding, polishing, gouging, incising, and notching, their adaptation to the selected raw material, and the alternating application of different tools and motions. It also requires the ability to mentally visualize a volume in matter and create symmetries in a three-dimensional space. Unlike paintings, anchored to sites charged with symbolic meaning, Paleolithic carvings are representations made to be transported, curated, manipulated, and often hung on clothing [30]. In view of the above, the cognitive requirements and technical skills needed to produce and perpetuate painted, engraved and drawn representations, on the one hand, and sculptures, on the other hand, may vary considerably and justifies approaching the emergence of these practices independently. The bird figurine from Lingjing constitutes the first carving found at an East Asian Paleolithic site and it differs technologically and stylistically from previous and contemporaneous representations of avifauna found in Europe and Siberia. The earliest known statuettes, made of mammoth ivory and including a flying waterfowl, are found in the Aurignacian of the Swabian Jura [29,30]. They are dated to *c*. 40–38 ka BP. Few other three-dimensional carvings representing birds, made of teeth and antler, come from West European late Upper Paleolithic sites [94–97]. The only Paleolithic bird carvings from Asia are those found at Mal’ta and Buret’, two neighboring Siberian sites located west of Lake Baikal [98]. They mainly consist of pendants made of ivory and antler representing flying waterfowls. The Lingjing figurine is the only Paleolithic three-dimensional object carved in burnt bone and representing a bird standing on a pedestal. It is also the only Paleolithic carving for which, thanks to its exceptional state of preservation, the final stages of manufacture could be documented in detail.

## Supporting information

S1 Data Interactive 3D.pdf model of the Lingjing bird carving obtained by microtomography. (PDF)

S1 Video 3D model of the Lingjing bird with longitudinal and transverse sections showing the reticular fibrolamellar bone structure and vascularization pattern. (MP4)

## References

[1] Mellars P. Major issues in the emergence of modern humans. Curr Anthropol. 1989;30: 349–385. 10.1086/203755

[2] Gamble C. The Palaeolithic societies of Europe. Cambridge: Cambridge University Press; 1999.

[3] Mithen S. The prehistory of mind: The cognitive origins of art, religion and science. New York: Thames and Hudson; 1999.

[4] McBrearty S, Brooks AS. The revolution that wasn’t: A new interpretation of the origin of modern human behavior. J Hum Evol. 2000;39: 453–563. 10.1006/jhev.2000.0435 11102266

[5] d’Errico F, Stringer CB. Evolution, revolution or saltation scenario for the emergence of modern cultures? Philos Trans R Soc B Biol Sci. 2011;366: 1060–1069. 10.1098/rstb.2010.0340 21357228 PMC3049097

[6] Colagè I, d’Errico F. Culture: The driving force of human cognition. Top Cogn Sci. 2018; Forthcoming. 10.1111/tops.12372 30033618

[7] Galway-Witham J, Cole J, Stringer C. Aspects of human physical and behavioural evolution during the last 1 million years. J Quat Sci. 2019;34: 355–378. 10.1002/jqs.3137

[8] Pettitt P. The Neanderthal dead. Exploring mortuary variability in Middle Palaeolithic Eurasia. Farming. 2002;4: 1–26.

[9] Soressi M, d’Errico F. Pigments, gravures, parures: Les comportements symboliques controversés des Néandertaliens In: Vandermeersch B, Maureille B, editors. Les Néandertaliens: Biologie et cultures. Paris: Éditions du CTHS; 2007 pp. 297–309.

[10] Henshilwood CS, d’Errico F, editors. Homo Symbolicus The dawn of language, imagination and spirituality. Amsterdam: John Benjamins Publishing Company; 2011.

[11] Henshilwood CS, d’Errico F, van Niekerk KL, Coquinot Y, Jacobs Z, Lauritzen S-E, et al A 100,000-year-old ochre-processing workshop at Blombos Cave, South Africa. Science. 2011;334: 219–222. 10.1126/science.1211535 21998386

[12] Vanhaeren M, d’Errico F, van Niekerk KL, Henshilwood CS, Erasmus RM. Thinking strings: Additional evidence for personal ornament use in the Middle Stone Age at Blombos Cave, South Africa. J Hum Evol. 2013;64: 599–517. 10.1016/j.jhevol.2013.02.001 23498114

[13] Joordens JCA, d’Errico F, Wesselingh FP, Munro S, de Vos J, Wallinga J, et al *Homo erectus* at Trinil on Java used shells for tool production and engraving. Nature. 2015;518: 228–231. 10.1038/nature13962 25470048

[14] Henshilwood CS, d’Errico F, van Niekerk KL, Dayet L, Queffelec A, Pollarolo L. An abstract drawing from the 73,000-year-old levels at Blombos Cave, South Africa. Nature. 2018;562: 115 10.1038/s41586-018-0514-3 30209394

[15] Li Z, Doyon L, Li H, Wang Q, Zhang Z, Zhao Q, et al Engraved bones from the archaic hominin site of Lingjing, Henan Province. Antiquity. 2019;93: 886–900. 10.15184/aqy.2019.81

[16] Pitarch Martí A, d’Errico F, Turq A, Lebraud E, Discamps E, Gravina B. Provenance, modification and use of manganese-rich rocks at Le Moustier (Dordogne, France). PLOS ONE. 2019;14: e0218568 10.1371/journal.pone.0218568 31314755 PMC6636720

[17] Aubert M, Brumm A, Ramli M, Sutikna T, Saptomo EW, Hakim B, et al Pleistocene cave art from Sulawesi, Indonesia. Nature. 2014;514: 223–227. 10.1038/nature13422 25297435

[18] Aubert M, Setiawan P, Oktaviana AA, Brumm A, Sulistyarto PH, Saptomo EW, et al Palaeolithic cave art in Borneo. Nature. 2018;564: 254–257. 10.1038/s41586-018-0679-9 30405242

[19] Aubert M, Lebe R, Oktaviana AA, Tang M, Burhan B, Hamrullah, et al Earliest hunting scene in prehistoric art. Nature. 2019;576: 442–445. 10.1038/s41586-019-1806-y 31827284

[20] Hoffmann DL, Standish CD, García-Diez M, Pettitt PB, Milton JA, Zilhão J, et al U-Th dating of carbonate crusts reveals Neandertal origin of Iberian cave art. Science. 2018;359: 912–915. 10.1126/science.aap7778 29472483

[21] Aubert M, Brumm A, Huntley J. Early dates for ‘Neanderthal cave art’ may be wrong. J Hum Evol. 2018;125: 215–217. 10.1016/j.jhevol.2018.08.004 30173883

[22] Pearce DG, Bonneau A. Trouble on the dating scene. Nat Ecol Evol. 2018;2: 925–926. 10.1038/s41559-018-0540-4 29632350

[23] Slimak L, Fietzke J, Geneste J-M, Ontañón R. Comment on “U-Th dating of carbonate crusts reveals Neandertal origin of Iberian cave art”. Science. 2018;361 10.1126/science.aau1371 30237321

[24] White R, Bosinski G, Bourrillon R, Clottes J, Conkey MW, Rodriguez SC, et al Still no archaeological evidence that Neanderthals created Iberian cave art. J Hum Evol. 2019; 102640. 10.1016/j.jhevol.2019.102640

[25] Aubert M, Brumm A, Taçon PSC. The timing and nature of human colonization of Southeast Asia in the Late Pleistocene: A rock art perspective. Curr Anthropol. 2017;58: S553–S566. 10.1086/694414

[26] Hoffmann DL, Standish CD, Pike AWG, García-Diez M, Pettitt PB, Angelucci DE, et al Dates for Neanderthal art and symbolic behaviour are reliable. Nat Ecol Evol. 2018;2: 1044–1045. 10.1038/s41559-018-0598-z 29942018

[27] Hoffmann DL, Standish CD, García-Diez M, Pettitt PB, Milton JA, Zilhão J, et al Response to Comment on “U-Th dating of carbonate crusts reveals Neandertal origin of Iberian cave art”. Science. 2018;362 10.1126/science.aau1736 30309914

[28] Hoffmann DL, Standish CD, García-Diez M, Pettitt PB, Milton JA, Zilhão J, et al Response to Aubert et al.’s reply ‘Early dates for “Neanderthal cave art” may be wrong’ [J. Hum. Evol. 125 (2018), 215–217]. J Hum Evol. 2019;135: 102644. 10.1016/j.jhevol.2019.102644 30173883

[29] Conard NJ. Palaeolithic ivory sculptures from southwestern Germany and the origins of figurative art. Nature. 2003;426: 830–832. 10.1038/nature02186 14685236

[30] Dutkiewicz E, Wolf S, Floss H, Conard NJ. Les objets en ivoire du Jura souabe. L’Anthropologie. 2018;122: 447–468. 10.1016/j.anthro.2018.05.003

[31] Gaut B, Livingston P, editors. The creation of art: New essays in philosophical æsthetics. Cambridge: Cambridge University Press; 2003.

[32] Chen C. Preliminary exploration of the typology and technology of microcore in China—Also of the culture relationship between Northeast Asia and Northwestern North America. Acta Anthropol Sin. 1983;2: 331–346.

[33] Li Z, Wu X, Zhou L, Liu W, Gao X, Nian X, et al Late Pleistocene archaic human crania from Xuchang, China. Science. 2017;355: 969–972. 10.1126/science.aal2482 28254945

[34] Nian XM, Zhou LP, Qin JT. Comparisons of equivalent dose values obtained with different protocols using a lacustrine sediment sample from Xuchang, China. Radiat Meas. 2009;44: 512–516. 10.1016/j.radmeas.2009.06.002

[35] Li H, Li Z, Lotter MG, Kuman K. Formation processes at the early Late Pleistocene archaic human site of Lingjing, China. J Archaeol Sci. 2018;96: 73–84. 10.1016/j.jas.2018.05.004

[36] Trinkaus E, Wu X-J. External auditory exostoses in the Xuchang and Xujiayao human remains: Patterns and implications among eastern Eurasian Middle and Late Pleistocene crania. PLOS ONE. 2017;12: e0189390 10.1371/journal.pone.0189390 29232394 PMC5726651

[37] Doyon L, Li H, Li Z, Wang H, Zhao Q. Further evidence of organic soft hammer percussion and pressure retouch from Lingjing (Xuchang, Henan, China). Lithic Technol. 2019;44: 100–117. 10.1080/01977261.2019.1589926

[38] Doyon L, Li Z, Li H, d’Errico F. Discovery of *circa* 115,000-year-old bone retouchers at Lingjing, Henan, China. PLOS ONE. 2018;13: e0194318 10.1371/journal.pone.0194318 29529079 PMC5847243

[39] van Kolfschoten T, Li Z, Wang H, Doyon L. The Middle Palaeolithic site of Lingjing (Xuchang, Henan, China): Preliminary new results. Analecta Praehist Leiden. 2020;50: 21–28.

[40] Li Z, Kunikita D, Kato S. Early pottery from the Lingjing site and the emergence of pottery in northern China. Quat Int. 2017;441: 49–61. 10.1016/j.quaint.2016.06.017

[41] Li Z, Xing Y, Mu J, Wu X, Li Y, Kato S. Report of the excavation of microlithic implements on the 5th layer of Lingjing, Xuchang Man site from 2008 to 2013. Huaxia Kaogu / Huaxia Archaeol. 2018;2018: 3–33.

[42] Li Z, Ma H. Techno-typological analysis of the microlithic assemblage at the Xuchang Man site, Lingjing, central China. Quat Int. 2016;400: 120–129. 10.1016/j.quaint.2015.08.065

[43] Li Z, Li Y, Kato S. Observations on microblade core technologies from lever 5 of the Xuchang Man site, Lingjing. Acta Anthropol Sin. 2014;33: 285–303.

[44] Bronk Ramsey C. Methods for summarizing radiocarbon datasets. Radiocarbon. 2017;59: 1809–1833. 10.1017/RDC.2017.108

[45] Reimer PJ, Bard E, Bayliss A, Beck JW, Blackwell PG, Bronk Ramsey C, et al IntCla13 and Marine13 radiocarbon age calibration curves 0–50,000 years cal BP. Radiocarbon. 2013;55: 1869–1887.

[46] Snoeck C, Brock F, Schulting RJ. Carbon exchanges between bone apatite and fuels during cremation: Impact on radiocarbon dates. Radiocarbon. 2014;56: 591–602. 10.2458/56.17454

[47] Cohen DJ. The advent and spread of early pottery in East Asia: New dates and new considerations for the world’s earliest ceramic vessels. J Austronesian Stud. 2013;4: 55–92.

[48] Wu X, Zhang C, Goldberg P, Cohen D, Pan Y, Arpin T, et al Early pottery at 20,000 years ago in Xianrendong Cave, China. Science. 2012;336: 1696–1700. 10.1126/science.1218643 22745428

[49] Cohen DJ, Bar-Yosef O, Wu X, Patania I, Goldberg P. The emergence of pottery in China: Recent dating of two early pottery cave sites in South China. Quat Int. 2017;441: 36–48. 10.1016/j.quaint.2016.08.024

[50] Wang Y, Zhang S, Gu W, Wang S, He J, Wu X, et al Lijiagou and the earliest pottery in Henan Province, China. Antiquity. 2015;89: 273–291. 10.15184/aqy.2015.2

[51] Provenzano N. Techniques et procédés de fabrication des industries osseuses terramaricoles de l’Âge du bronze. In: Julien M, Averbouh A, Ramseyer D, Bellier C, Buisson D, Cattelain P, et al., editors. Préhistoire d’os. Aix-en-Provence: Université de Provence; 1999. pp. 273–288.

[52] Averbouh A, Provenzano N. Propositions pour une terminologie du travail préhistorique des matières osseuses: I—Les techniques. Préhistoire Anthropol Méditerranéennes. 1998;7–8: 5–25.

[53] Averbouh A, Bergouën R, Clottes J. Technique et économie du travail du bois de cervidé chez les Magdaléniens d’Enlène (Montesquieu-Avantès, Ariège): Vers l’identification d’un cycle saisonnier de production? In: Julien M, Averbouh A, Ramseyer D, Bellier C, Buisson D, Cattelain P, et al., editors. Préhistoire d’os. Aix-en-Provence: Université de Provence; 1999. pp. 289–318.

[54] David É. Transformation des matières dures d’origine animale dans le Mésolithique de l’Europe du Nord. In: Ramseyer D, editor. Fiches typologiques de l’industrie osseuse préhistorique; Cahier XI Matières et techniques. Paris, France: Éditions Société Préhistorique Française; 2004. pp. 113–149.

[55] Le Dosseur G. Sens et contre sens. Réflexions concernant l’orientation d’un geste technique observé sur des objets en matière osseuse du Levant. Préhistoires Méditerranéennes. 2003; 115–127.

[56] Sidéra I, Legrand A. Tracéologie fonctionnelle des matières osseuses: une méthode. Bull Société Préhistorique Fr. 2006;103: 291–304.

[57] Gates St-Pierre C, Walker RB, editors. Bone as tools: Current methods and interpretations in worked bone studies. Oxford: Oxbow Books; 2007.

[58] Buc N. Experimental series and use-wear in bone tools. J Archaeol Sci. 2011;38: 546–557. 10.1016/j.jas.2010.10.009

[59] LeMoine GM. Use wear on bone and antler tools from the Mackenzie Delta, Northwest Territories. Am Antiq. 1994;59: 316–334. 10.2307/281935

[60] d’Errico F. La vie sociale de l’art mobilier paléolithique. Manipulation, transport, suspension des objets en os, bois de cervidés, ivoire. Oxf J Archaeol. 1993;12: 145–174. 10.1111/j.1468-0092.1993.tb00289.x

[61] Zhang S, d’Errico F, Backwell LR, Zhang Y, Chen F, Gao X. Ma’anshan cave and the origin of bone tool technology in China. J Archaeol Sci. 2016;65: 57–69. 10.1016/j.jas.2015.11.004

[62] Zhang S, Doyon L, Zhang Y, Gao X, Chen F, Guan Y, et al Innovation in bone technology and artefact types in the late Upper Palaeolithic of China: Insights from Shuidonggou Locality 12. J Archaeol Sci. 2018;93: 82–93. 10.1016/j.jas.2018.03.003

[63] Orme CDL, Davies RG, Olson VA, Thomas GH, Ding T-S, Rasmussen PC, et al Global patterns of geographic range size in birds. PLOS Biol. 2006;4: e208 10.1371/journal.pbio.0040208 16774453 PMC1479698

[64] Cracraft J, Barker FK. Passerine birds (Passeriformes) In: Hedges SB, Kumar S, editors. The timetree of life. New York: Oxford University Press; 2009 pp. 423–431. http://www.timetree.org/public/data/pdf/Cracraft2009Chap61.pdf.

[65] Turner–Walker G, Syversen U. Quantifying histological changes in archaeological bones using BSE–SEM image analysis. Archaeometry. 2002;44: 461–468. 10.1111/1475-4754.t01-1-00078

[66] Bell LS. Palaeopathology and diagenesis: An SEM evaluation of structural changes using backscattered electron imaging. J Archaeol Sci. 1990;17: 85–102. 10.1016/0305-4403(90)90016-X

[67] Bell LS, Boyde A, Jones SJ. Diagenetic alteration to teeth in situ illustrated by backscattered electron imaging. Scanning. 1991;13: 173–183. 10.1002/sca.4950130204

[68] Fairbridge RW. Diagenetic minerals In: Frye K, editor. The encyclopedia of Mineralogy. Encyclopedia of Earth sciences series. Boston, MA: Springer US; 1981 pp. 121–123.

[69] Hedges REM. Bone diagenesis: an overview of processes. Archaeometry. 2002;44: 319–328. 10.1111/1475-4754.00064

[70] Francillon-Vieillot H, Buffrenil VD, Castanet J, Geraudie J, Meunier FJ, Sire JY, et al Microstructure and mineralization of vertebrate skeletal tissues In: Carter JG, editor. Skeletal biomineralization: Patterns, processes and evolutionary trends. New York: Van Nortrand Reinhold; 1990 pp. 471–530.

[71] de Margerie E, Cubo J, Castanet J. Bone typology and growth rate: testing and quantifying ‘Amprino’s rule’ in the mallard (*Anas platyrhynchos*). C R Biol. 2002;325: 221–230. 10.1016/s1631-0691(02)01429-4 12017770

[72] Currey JD. Bones: Structure and mechanics. Oxford: Princeton University Press; 2002 http://library.wur.nl/WebQuery/clc/1854910.

[73] Enlow DH, Brown SO. A comparative histological study of fossil and recent bone tissues. Part II. Tex J Sci. 1957;9: 186–204.

[74] De Ricqles A, Meunier FJ, Castanet J, Francillon-Vieillot H. Comparative microstructure of bone In: Hall BK, editor. Bone, Vol 3: Bone matrix and bone specific products. Boston: CRC Press; 1991 pp. 1–78.

[75] Locke M. Structure of long bones in mammals. J Morphol. 2004;262: 546–565. 10.1002/jmor.10282 15376271

[76] Fernández-Jalvo Y, Andrews P. Atlas of taphonomic identifications. New York: Springer Dordrecht; 2016.

[77] Bradtmiller B, Buikstra JE. Effects of burning on human bone microstructure: A preliminary study. J Forensic Sci. 1984;29: 535–540. 10.1520/JFS11701J 6726157

[78] Castillo RF, Ubelaker DH, Acosta JAL, de la Fuente GAC. Effects of temperature on bone tissue. Histological study of the changes in the bone matrix. Forensic Sci Int. 2013;226: 33–37. 10.1016/j.forsciint.2012.11.012 23287528

[79] Castillo RF, Ubelaker DH, Acosta JAL, de la Rosa RJE, Garcia IG. Effect of temperature on bone tissue: Histological changes. J Forensic Sci. 2013;58: 578–582. 10.1111/1556-4029.12093 23458344

[80] Cattaneo C, DiMartino S, Scali S, Craig OE, Grandi M, Sokol RJ. Determining the human origin of fragments of burnt bone: A comparative study of histological, immunological and DNA techniques. Forensic Sci Int. 1999;102: 181–191. 10.1016/s0379-0738(99)00059-6 10464934

[81] Ellingham STD, Thompson TJU, Islam M, Taylor G. Estimating temperature exposure of burnt bone—A methodological review. Sci Justice. 2015;55: 181–188. 10.1016/j.scijus.2014.12.002 25934370

[82] Hanson M, Cain CR. Examining histology to identify burned bone. J Archaeol Sci. 2007;34: 1902–1913. 10.1016/j.jas.2007.01.009

[83] Huisman H, Ismail-Meyer K, Sageidet BM, Joosten I. Micromorphological indicators for degradation processes in archaeological bone from temperate European wetland sites. J Archaeol Sci. 2017;85: 13–29. 10.1016/j.jas.2017.06.016

[84] Imaizumi K. Forensic investigation of burnt human remains. Res Rep Forensic Med Sci. 2015;5: 67–74. 10.2147/RRFMS.S75141

[85] Mulhern DM, Ubelaker DH. Differences in osteon banding between human and nonhuman bone. J Forensic Sci. 2001;46: 220–222. 10.1520/JFS14952J 11305421

[86] Walker PL, Miller KWP, Richman R. 7—Time, temperature, and oxygen availability: An experimental study of the effects of environmental conditions on the color and organic content of cremated bone In: Schmidt CW, Symes SA, editors. The analysis of burned human remains. San Diego: Academic Press; 2008 pp. 129–135.

[87] Mitchell WJT. Representation In: Lentricchia F, McLaughlin T, editors. Critical terms for literary study. Chicago: The University of Chicago Press; 1995 pp. 11–22.

[88] Vukcevich M. Representation. The Chicago School of Media Theory RSS. Chicago: Chicago School of Media Theory; 2002. http://csmt.uchicago.edu/glossary2004/representation.htm.

[89] Shi X. Issues on bird images and bird worship of the ancient cultures in the eastern coastal area and southeast China In: Tian C, Shi X, editors. Collection of papers on Chinese prehistoric cultures. Beijing: Wenwu Press; 1989 pp. 234–266.

[90] Liu D. Art history and ancient civilization. Taibei: Yuncheng Culture Industrial Co., Ltd; 1994.

[91] Hopkins R. Painting, sculpture, sight, and touch. Br J Æsthet. 2004;44: 149–166. 10.1093/bjaesthetics/44.2.149

[92] Seitamaa-Hakkarainen P, Huotilainen M, Mäkelä M, Groth C, Hakkarainen K. How can neuroscience help understand design and craft activity? The promise of cognitive neuroscience in design studies. FormAkademisk—Forskningstidsskrift Des Og Des. 2016;9 10.7577/formakademisk.1478

[93] Cialone C, Tenbrink T, Spiers HJ. Sculptors, architects, and painters conceive of depicted spaces differently. Cogn Sci. 2018;42: 524–553. 10.1111/cogs.12510 28656679 PMC5873447

[94] Menéndez Fernández M. Excavaciones arqueologicas en la Cueva del Buxu (Cardes. Cangas de Onis). Excavaciones Arqueol En Astur. 1992;1992: 69–74.

[95] Fortea Pérez FJ. Abrigo de la Viña. Informe de la campañas 1980–1986. Excavaciones Arqueol En Astur. 1986;1986: 55–68.

[96] Ortega I, Rios-Garaizar J, Garate Maidagan D, Arizaga J, Bourguignon L. A naturalistic bird representation from the Aurignacian layer at the Cantalouette II open-air site in southwestern France and its relevance to the origins of figurative art in Europe. J Archaeol Sci Rep. 2015;4: 201–209. 10.1016/j.jasrep.2015.09.009

[97] Buisson D, Pinçon G. Nouvelle lecture d’un galet gravé de Gourdan et essai d’analyse des figurations d’oiseaux dans l’art paléolithique français. Antiq Natl. 1986;18–19: 75–90.

[98] Abramova ZA. L’art paléolithique d’Europe orientale et de Sibérie. Grenoble: Jérôme Millon; 1995.

