# Supplementary material for: A Paleolithic bird figurine from the Lingjing site, Henan, China
Source: PLoS One. 2020 Jun 10;15(6):e0233370. doi: 10.1371/journal.pone.0233370 (PMC7286485; doi:10.1371/journal.pone.0233370)
Supplement: S1 Data — (PDF) [file pone.0233370.s001.pdf]

LingJing Bird

3D surface of the LingJing bird - Right click to visualize full screen.
